# Cortical Plasticity after Cochlear Implantation

**DOI:** 10.1155/2013/318521

**Published:** 2013-11-26

**Authors:** B. Petersen, A. Gjedde, M. Wallentin, P. Vuust

**Affiliations:** ^1^Center for Functionally Integrative Neuroscience, Aarhus University Hospital, Nørrebrogade 44, Building 10G 6th, 8000 Aarhus C, Denmark; ^2^Royal Academy of Music, Skovgaardsgade 2a, 8000 Aarhus C, Denmark; ^3^Department of Neuroscience and Pharmacology, University of Copenhagen, Blegdamsvej 3, 2200 København N, Denmark; ^4^Center for Semiotics, Aarhus University, Building 1485, Office 620, Jens Chr. Skous Vej, 8000 Aarhus C, Denmark

## Abstract

The most dramatic progress in the restoration of hearing takes place in the first months after cochlear implantation. To map the brain activity underlying this process, we used positron emission tomography at three time points: within 14 days, three months, and six months after switch-on. Fifteen recently implanted adult implant recipients listened to running speech or speech-like noise in four sequential PET sessions at each milestone. CI listeners with postlingual hearing loss showed differential activation of left superior temporal gyrus during speech and speech-like stimuli, unlike CI listeners with prelingual hearing loss. Furthermore, Broca's area was activated as an effect of time, but only in CI listeners with postlingual hearing loss. The study demonstrates that adaptation to the cochlear implant is highly related to the history of hearing loss. Speech processing in patients whose hearing loss occurred after the acquisition of language involves brain areas associated with speech comprehension, which is not the case for patients whose hearing loss occurred before the acquisition of language. Finally, the findings confirm the key role of Broca's area in restoration of speech perception, but only in individuals in whom Broca's area has been active prior to the loss of hearing.

## 1. Introduction

The cochlear implant (CI) transforms acoustic signals from the environment into electric impulses, which are then used to stimulate intact fibers of the auditory nerve. With this treatment, individuals with profound hearing loss (HL) are given the opportunity to gain or regain the sense of hearing. Current technology and speech processing strategies allow many CI recipients to achieve impressive accuracy in open-set speech recognition, and the CI is arguably the most effective neural prosthesis ever developed [1–3]. However, the success of the outcome depends both on duration of deafness prior to implantation [4, 5] and on the onset of deafness before (prelingually) [4–7] or after (postlingually) [8] critical stages in the acquisition of language. In many cases, the greatest gains of performance occur in the first three months of use [9–11]. The dramatic improvements following implantation not only demonstrate the efficiency of the CI technology, but also point to the role of cortical plasticity as a means to reactivate brain function. 

Plasticity is a term used to describe the reorganization of the central nervous system by means of synaptic changes and rewiring of neural circuits. In cases of cochlear implantation, neural plasticity associated with deprivation of auditory input and adaptation to the absence of stimuli is of particular interest. Reduced input to the brain from impaired auditory pathways results in significant changes in the central auditory system [12] and is accompanied by a recruitment of deprived cortices in response to input from the intact senses [13–17]. When auditory input to the brain is reintroduced, this novel auditory experience may itself induce additional plasticity [18]. The sensory reafferentation provided by the CI thus offers a unique opportunity to study the effects of preceding deafness on functional brain organization. 

In normal-hearing (NH) adults, language processing is associated with extensive frontal activation in the left cerebral hemisphere, including the anterior (Brodmann's Areas (BA) 45 and 47) and posterior (BA 44 and 45) parts of the left inferior frontal gyrus (LIFG), the latter often referred to as Broca's area [19, 20]. Traditionally, this area is mainly assigned an expressive language function, but several studies show a relationship between the perception of language and left frontal activity, both when stimuli are presented aurally [21–24] and visually [24–28]. Neuroimaging experiments comparing auditory responses of CI users and normal-hearing control participants, while listening to speech or complex nonspeech, generally reveal bilateral activity in the primary and secondary auditory cortices, including both superior and middle temporal gyri [12, 29–34]. One consistent outcome of these studies is the more dominant right temporal activity of CI users listening to speech, that is, the observation of more bilateral activity than would be expected on the basis of the classical presumption of left-lateralized activity of language processing in normal-hearing [35]. However, in these studies, activation of other classic language regions such as Broca's area was not a consistent finding. Naito and colleagues found Broca's area to be activated only when the CI participants silently repeated sentences [31, 36]. Mortensen et al. [37] compared brain activity in experienced CI users according to their levels of speech comprehension performance. They found that, unlike CI users with low speech comprehension, single words and speech yielded raised activity in the left inferior prefrontal cortex (LIPC) in CI users with excellent speech perception. 

Some observed activations outside the classic language areas, including anterior cingulate, parietal regions, and left hippocampus, have been attributed to nonspecific attentional mechanisms and memory in CI users [31, 32]. Furthermore, some studies have reported convincing evidence of visual activity in response to auditory stimuli or auditory activity in response to visual stimuli in CI users. Although much debate about the identity of the brain systems that are changed and the mechanisms that mediate these changes exists, the general belief is that this cross-modal reorganization is associated with the strong visual speech-reading skills developed by CI users during the period of deafness, which are maintained or even improved after cochlear implantation, despite progressive recovery of auditory function [5, 11, 33, 38–41]. The possible reasons for these mixed results may include differences in experimental paradigms, small sample sizes, heterogeneous populations, variance in statistical thresholds, and a lack of longitudinal control of plasticity.

With the present study, we tested the cortical mechanisms underlying the restoration of hearing and speech perception in the first six-month period following implant switch-on. We expected to see inactive neuronal pathways reactivated in CI recipients, within three to six months of switch-on, and engagement of cortical areas resembling those of normal-hearing control participants. Furthermore, in previous findings, notwithstanding, we expected to see Broca's area involved in speech perception. Finally, we expected to see a difference in the progress of adaptation and the involvement of cortical areas between CI users with postlingual hearing loss and CI users with prelingual hearing loss.

## 2. Materials and Methods

### 2.1. Participants

Over the course of two years, patients who were approved for implantation were contacted by mail and invited to take part in the research project, including positron emission tomography (PET) and speech perception measures. From a total of 41 patients, 15 accepted and were included in the study (6 women, 9 men, *M*_age_ = 51.8, age, range: 21–73 years). All participants were unilaterally implanted. Four participants had a prelingual onset of HL, indicated by their estimated age at onset of deafness and main use of signed language as communicative strategy. The remaining 11 participants had a postlingual or progressive onset of HL, as indicated by their main use of residual hearing, supported by lip-reading. In accordance with local practice, all CI participants followed standard aural/oral therapy for six months in parallel with the study. The therapy program includes weekly one-hour individually adapted sessions and trains speech perception and articulation.  Table 1 lists the demographic and clinical data for the 15 participants.

### 2.2. Normal-Hearing Reference

To obtain a normal-hearing reference, we recruited a group of NH adults (4 women, 2 men, *M*_age_ = 54.29 years, age range: 47–64 years) for a single PET/test session. All NH participants met the criteria for normal-hearing by passing a full audiometric test.

All participants were right-handed Danish speakers and gender was not considered important [42].

### 2.3. Ethical Approval

The study was conducted at the PET center, Aarhus University Hospital, Denmark, in accordance with the Declaration of Helsinki, and approved by the Research Ethics Committee of the Central Denmark Region. Informed consent was obtained from all participants.

### 2.4. Design

NH participants underwent PET once, while CI participants were tested consecutively at three points of time: (1) within 14 days after switch-on of the implant (baseline, BL), (2) after three-months (midpoint, MP), and (3) after six months (endpoint, EP). For purposes of analysis, two subgroups were identified as (1) the postlingual (POST) HL subgroup (*N* = 11) and (2) the prelingual (PRE) HL subgroup (*N* = 4).

### 2.5. Behavioral Measures

We assessed the participants' speech perception progress by the Hagerman speech perception test [43]. The Hagerman test is an open-set test which presents sentences organized in lists of ten. The sentences have identical name-verb-number-adjective-noun structures, which the participant is required to repeat. Normally, the test is presented in background noise. However, considering the participants' inexperience with the implant, we removed the background noise. The participants were given one training list with feedback and two trial lists without feedback (max. score = 100 pts.). Sound was played back on a laptop computer through an active loudspeaker (Fostex 6301B, Fostex Company, Japan) placed in front of the participant. The stimuli were presented at 65 dB sound pressure level (SPL), and CI users were instructed to use their preferred CI settings during the entire test session and to adjust their processors to a comfortable loudness level. Furthermore, participants who used a hearing aid were instructed to turn it off and leave it plugged in. The CI participants performed the Hagerman tests in separate sessions at the same milestones as selected for PET data acquisition (BL, MP, and EP). Different lists were used at the three times of testing. The NH group performed a single Hagerman test along with their PET scan session.

To identify effects of time, we performed a repeated measures analysis of variance. Due to nonnormal distributions in the data, this was done using a nonparametric Friedman's ANOVA in the POST and PRE subgroups separately. Post hoc tests and between-group analyses were performed by Mann-Whitney nonparametric tests and Bonferroni corrected at alpha 0.016. The HAG data were analyzed in SPSS and plotted with Sigmaplot for Windows 11.0 (Systat Software Inc.).

### 2.6. Apparatus and Stimuli

*MRI.* A high-resolution T1-weighted MR scan was acquired prior to PET scanning. In the case of CI participants, this was performed preoperatively. 

*Stimuli.* All participants were examined in 2 conditions: (1) multitalker babble (BAB) from multiple simultaneous speakers with a complexity close to that of speech and perceived by the listeners as speech-like but devoid of meaning [44] and (2) “running” speech (RS), narrating the history of a familiar geographical locality at the rate of 142 words per minute, generated in Danish by a standard female voice [45]. The stimuli were played back on a laptop computer in the freeware sound editor software Audacity (http://audacity.sourceforge.net/) and delivered directly from the computer's headphone jack to the external input port of the implant speech processor. Bimodally aided participants removed their hearing aid and were fitted with an earplug in the nonimplanted ear during the tomography. These measures were taken to preclude background noise and possible cross-talk from the contralateral ear. The NH participants listened to the stimuli binaurally through a pair of headphones (AKG, K 271). All stimuli were presented at the most comfortable level. To define this level, participants were exposed to the two stimuli once before the tomography. In the tomograph, prior to bolus injection, participants had no information about the nature of the next stimulus, but they were instructed to listen attentively in all cases. After each of the four scans, participants were required to describe what they had heard and, if possible, review the content of the narration. 

*PET.* Positron emission tomography (PET) is a molecular imaging method that yields brain activity, by means of detecting changes in regional cerebral blood flow (rCBF). This is done by computing and comparing the spatial distributions of the uptake of a blood flow tracer. PET measurements are generally limited with respect to spatial and temporal resolution and the invasiveness of the procedure, which requires injection of oxygen-15-labelled water. In this case, however, anatomical and temporal specificity could not have been improved by using functional magnetic resonance imaging (fMRI), as the auditory implants are not MRI compatible. In addition, PET is an almost noiseless imaging modality, which is useful for both CI participants and for the study of speech. Finally, because only the head of the participant is positioned in the tomograph, compared with the whole body imposition of fMRI, it is possible to communicate visually with the participant during tomography.

We measured raised or reduced cerebral activity as the change of the brain uptake of H_2_^15^O oxygen-15-labeled water, which matches the distribution of cerebral blood flow (CBF), using an ECAT EXACT HR 47 Tomograph (Siemens/CTI). Emission scans were initiated at 60,000 true counts per second after repeated intravenous bolus injections of doses of tracer with an activity of 500 MBq (13.5 mCi). The tomography took place in a darkened room with participants' eyes closed.

The babble and running speech conditions were duplicated, generating a total of four tomography sessions. The uptake lasted 90 seconds (single frame) at intervals of 10 min. Each frame registered 47 3.1 mm sections of the brain. After correction for scatter and measured attenuation, each PET frame was reconstructed with filtered back projection and smoothed with a postreconstruction 10 mm Gaussian filter resulting in a resolution of 11 mm full-width-at-half-maximum (FWHM).

### 2.7. Restrictions

Rules of regulation mean that participants who volunteer for scientific experiments may receive a total maximum radiation of 6 millisieverts (mSv) within one year. Here, the total radiation dose administered over the three times of scanning was approximately 5.58 mSv. Due to these restrictions, no preoperative baseline scans could be acquired. 

### 2.8. Image Preprocessing

Participants' MR images were co-registered to an MR template averaged across 85 individual MR scans in Talairach space [46], using a combination of linear and nonlinear transformations [47]. Each summed PET emission recording was linearly coregistered to the corresponding MR image using automated algorithms. To smooth the PET images for individual anatomical differences and variation in gyral anatomy, images were blurred with a Gaussian filter resulting in final 14 mm at full-width half-maximum (FWHM) isotropic resolution.

### 2.9. Data Analysis

All images were processed using Statistical Parametric Mapping 8 (SPM8; Wellcome Neuroimaging Department, UK, (http://www.fil.ion.ucl.ac.uk/spm/)). Local maxima of activation clusters were identified using the Montreal Neurological Institute (MNI) coordinate system and then cross-referenced with a standard anatomical brain coordinate atlas [46]. Differences in global activity were controlled using proportional normalization (gray matter average per volume). Significance threshold for task main effects was set to *P* < 0.05, family wise error (FWE) corrected for multiple comparisons. We tested the effect of side of implant and type of implant in a separate preanalysis. As we found neither main effects nor interactions with functional data involving these variables, we concluded that these factors had no significant effect on the results. They were thus not included in further analyses.

Three analyses were performed as described below.

*Analysis  1.* The first analysis identified the main effects of time, speech/babble contrast, and history of hearing loss (POST HL versus PRE HL) and possible interactions between these effects in the whole brain, across CI participants. This analysis was performed as a single SPM matrix in a factorial 3-way design with time, contrast, and subgroup as factors. To define a region-of-interest (ROI), we created a mask based on the main effect of contrast. 

*Analysis  2.* The second analysis identified possible main effects of contrast, time, and interactions between these factors in the inferior frontal gyrus (IFG), more specifically Broca's area (BA 44/45). This analysis was performed as two 2-way factorial analyses of the POST HL and the PRE HL subgroups separately. To define a region-of-interest (ROI), we created a mask based on bilateral inferior frontal gyri (Broca's region), including the putative Brodmann regions 44, 45, and 47 using the WFU pick-atlas [48]. 

*Analysis  3.* The third analysis investigated main effects of contrast and group (CI versus NH) at the CI baseline and possible interactions between these effects. This analysis was performed as a single SPM matrix in a factorial 2-way design with condition and group as factors. To define a ROI, we created a mask based on the main effect of contrast.

## 3. Results

### 3.1. Analysis  1

**Figure 1:**
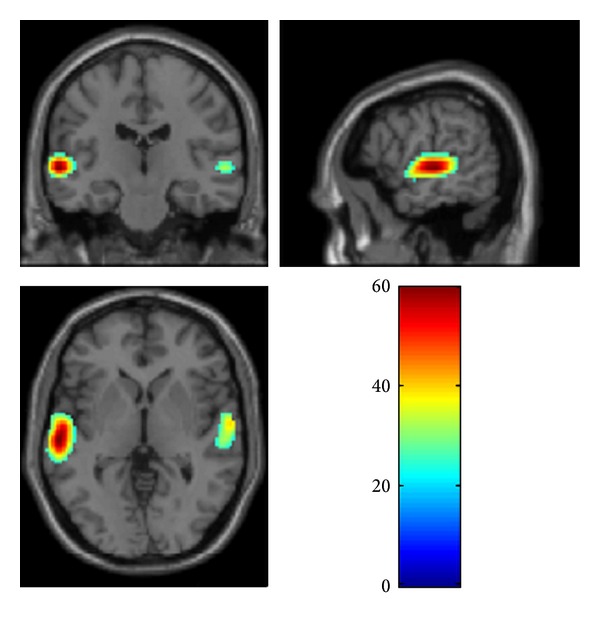
Activation map for main effect of contrast across subgroups in the whole brain analysis showing greater activity in superior temporal gyri (BA 21/22) during speech comprehension.

**Figure 2:**
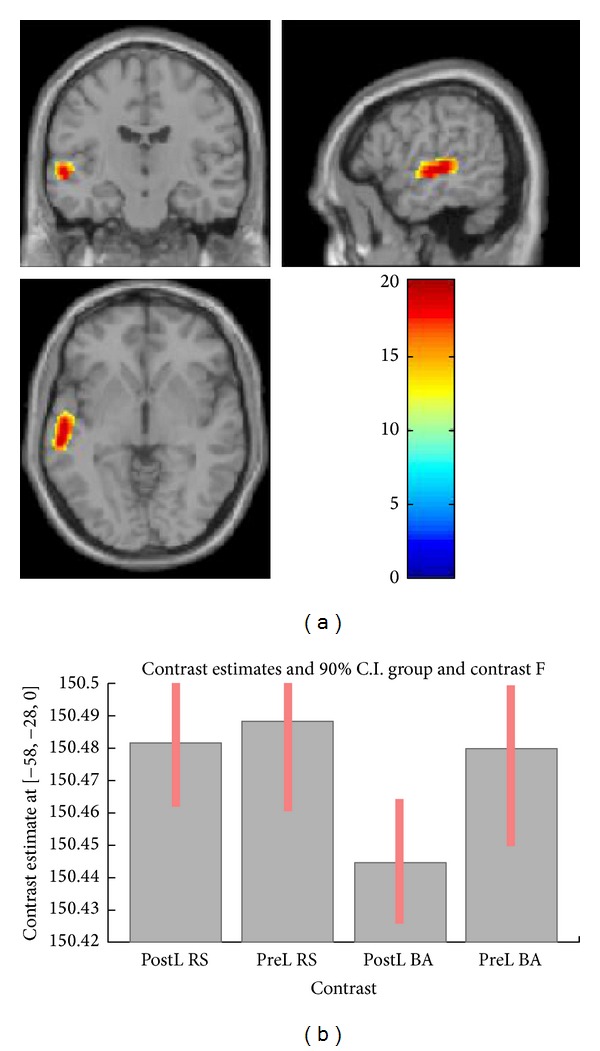
(a) Activation map for interaction between effect of contrast and effect of PRE/POST subgroup in the ROI analysis (L BA 21/22) showing greater activation during speech for the postlingual group. (b) Contrast estimates of conditions in the two subgroups showing a larger difference in the postlingual subgroup than in the prelingual subgroup. PostL: postlingual group; PreL: prelingual group; RS: running speech condition; BA: multitalker babble condition.

We found a main effect of speech/babble contrast across CI participants, regardless of subgroup, in bilateral superior temporal gyri (*F*(1,78) = 60.14). A *t*-test confirmed that the effect was driven by higher activity during running speech (Table 2; Figure 1). There was no significant main effect of time or group, nor any interaction between the effects. The ROI analysis revealed significant interaction between the effects of contrast and group in BA 21/22 in the left superior temporal gyrus (*F*(1,78) = 20.42) (Table 2; Figure 2). A plot of contrast estimates showed a difference between running speech and babble that was larger in the postlingual subgroup than in the prelingual subgroup (Figure 2).

### 3.2. Analysis 2

**Figure 3:**
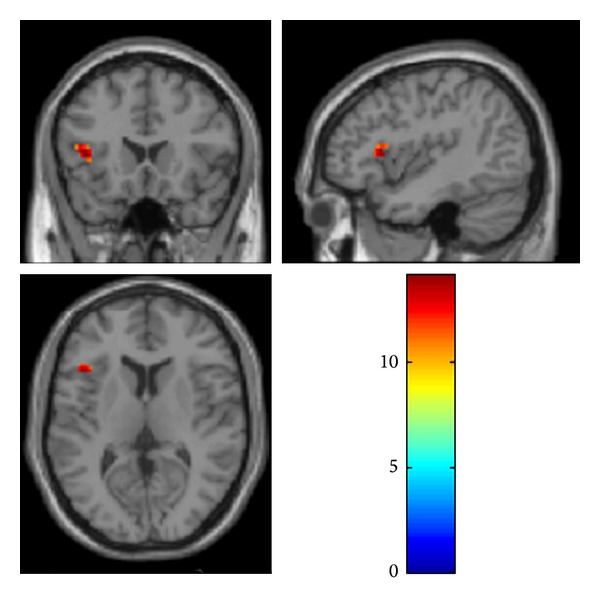
Activation map for main effect of time in the separate analysis of the POST HL group with ROI based on bilateral IFG showing activation of left IFG (Broca's area BA 45).

In the bilateral IFG ROI analysis, we found a main effect of speech/babble contrast in BA 47 in the postlingual subgroup. A *t*-test confirmed that the effect was driven by higher activity during running speech. Furthermore, we found a main effect of time in left IFG (Broca's area BA 45; *F*(2,60) = 14.19; *P* = 0.006, FWE corrected) (Figure 3), with no significant interaction between contrast and time. The prelingual subgroup had no main effects in the bilateral IFG ROI analysis (Table 3).

### 3.3. Analysis 3

We found a main effect of speech/babble contrast across the CI and NH groups bilaterally in superior temporal gyri, in the left middle temporal gyrus, and in the right inferior parietal lobule. *t*-tests showed that the superior temporal gyri bilaterally and the left middle temporal gyrus were more active during running speech, while the right inferior parietal lobule was more active during babble. We found a main effect of CI versus NH exclusively in the caudate nucleus. A *t*-test showed that this effect was due to higher activity of this area in the NH group than in the CI group. No interaction was found between the effect of contrast and the effect of group in whole-brain analysis (Table 4).

The ROI analysis based on the main effect of contrast yielded a main effect of CI versus NH in secondary auditory cortex including BA 22 in the right superior temporal gyrus. A *t*-test showed that this effect was due to higher activity of this area in the NH group than in the CI group. Furthermore, in the ROI analysis, we found an interaction between the effect of speech/babble contrast and the effect of group in the right inferior parietal lobule (Table 4).

### 3.4. Behavioral Measures

**Figure 4:**
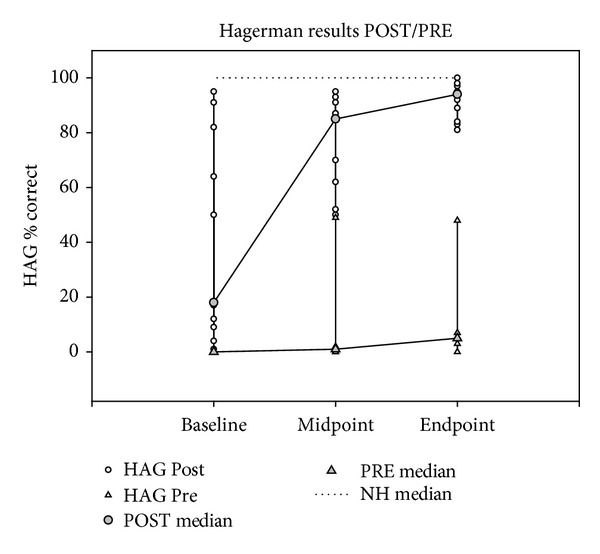
Mixed line and scatter plot showing individual and median Hagerman speech perception scores for the POST and the PRE subgroups at the three milestones. The dotted line represents the median score of the NH reference group.

We found a significant effect of time in the POST group (*χ* ^2^(2,2) = 19.62, *P* < 0.001), but not in the PRE group. Post hoc tests showed that the effect was driven by the 51.3 percentage points endpoint gain (Mann-Whitney *U* = 12.5, *P* = 0.002). Furthermore, the POST group scored significantly higher than the PRE group at all three points of measurement (BL: Mann-Whitney *U* = 0.000, *P* = 0.004; MP: Mann-Whitney *U* = 0.000, *P* = 0.004; EP: Mann-Whitney *U* = 0.000, *P* = 0.004). Ceiling performance (100% correct) was observed in all NH participants (Figure 4).

## 4. Discussion

In this study, we aimed to examine the plastic changes which underlie the recovery of hearing after cochlear implantation. Our results showed that across all CI recipients and all points of measurement, the bilateral middle and superior temporal gyri (including, more specifically, Brodmann areas 21 and 22) were significantly more active when participants listened to running speech than when they listened to multi-talker babble. Thus, on average, the auditory brain regions, known to be involved in the processing of complex auditory stimuli [49–51] displayed a clear distinction between speech-like noise and speech in recently implanted CI recipients. This confirms that both hemispheres are involved in the speech perception process, even during monaural auditory stimulation [29, 36, 52, 53].

Furthermore, the results showed a difference in the way CI recipients with postlingual hearing loss and recipients with prelingual hearing loss distinguish between speech and babble. The CI users with postlingual hearing loss displayed a greater activation during speech than during babble in BA 21 and 22 in the left superior temporal gyrus, indicating differential processing of the stimuli by the two subgroups. We speculate that the postlingually deaf listeners disengage attention when they are presented with the incomprehensible babble stimulus. This disengagement is then reflected in decreased temporal brain activity. In contrast, the prelingually deaf CI listeners may be equally attentive to the two stimuli, regardless of their nature, as reflected in undifferentiated activity.

This difference between postlingual and prelingual deafness was mirrored in the behavioral measures. The postlingual subgroup not only possessed a moderate level of speech perception at baseline (within 14 days after switch-on of the implant), but also made significant gains in performance, the majority of which occurred in the first three month period. In contrast, the prelingual subgroup had no baseline speech perception and only modest, if any, progress during the study period. This finding is consistent with expectation and implies an association between behavioral performance and brain activity related to the history of hearing loss. In prelingual deafness, the neuronal connections of the auditory pathways (e.g., measured as cortical auditory evoked potentials) may not be established in the appropriate time window of opportunity [54–56]. The subsequent electric stimulation at some time in adulthood may produce some hearing sensation, but the discrimination of sounds and time intervals remain defective [57, 58]. The findings are compatible with Naito et al. [29], who suggested that the reduced speech activation in prelingually deaf implant users could be explained by insufficient development of neuronal networks or their degeneration due to prolonged deafness. Follow-up studies in the present population may provide interesting insight into the degree to which speech perception progresses in the prelingual subgroup in the long term.

We found a main effect of time exclusively in Broca's area, and only in the postlingual subgroup. This is in line with recent studies showing that, in CI users, the activation of Broca's area during speech processing is negatively correlated with the duration of deafness and positively correlated with the progress in the restoration of speech comprehension [7, 8, 37, 59]. This indicates that the changes in the auditory recovery process are most profoundly manifested in this specific area, which is associated with speech perception and production. Surprisingly, we found no interaction between the speech/babble contrast and time. This suggests that the area becomes increasingly activated, regardless of whether the stimulus makes semantic sense or not, or is active in the distinction between sense and nonsense. However, the absence of difference between speech and babble in Broca's area may also be explained by an increasingly high activity at rest in CI patients, as reported by Strelnikov et al. [39].

### 4.1. Cochlear Implanted Participants at Baseline versus Normal-Hearing Participants

Our whole-brain analysis revealed a significant activation of bilateral superior temporal gyri and left middle temporal gyrus during speech across CI participants at baseline and NH individuals. This is partly consistent with Naito et al. [31], who, in addition to bilateral superior temporal gyri, found significant speech activation in the right middle temporal gyrus, the left posterior inferior frontal gyrus (Broca's area), and the left hippocampus in both normal participants and CI users. These further findings could be explained by the use of silence as contrast relative to the use of babble in the present study. Interestingly, even though the NH participants received unilateral stimulation (6 right; 6 left), the Naito study found no significant difference in any brain area in either NH participants or CI users between right ear stimulation and left ear stimulation.

The significant involvement of the right parietal lobule in the CI participants during babble suggests that, at this initial stage of the CI adaptation, CI listeners, unlike NH individuals, need to pay attention to the speech-like noise to determine its possible character [60]. The observation that during speech stimulation the NH participants involved the caudate nucleus more than the CI participants may be explained by a reduction of the effort needed by the NH participants to deal with the well-known task of receiving a message. The caudate nucleus is a part of the striatum, which subserves among other tasks the learning of slowly modulated skills or habits [61]. To the normal-hearing listener, the reception of auditory information is an every-day experience similar to following a known route; for example, see Wallentin et al. [23] for a similar argument. In contrast, to CI listeners, auditory stimuli are nonhabitual in the strongest sense of the word, thus relying on other sources of processing. Interestingly, Naito et al. [31] made a similar finding and speculated that though the caudate nucleus has been associated with various tasks ranging from sensorimotor tasks to pure thinking, the result may provide further evidence that the area has cognitive function and shows increased activity along with the increased activity in cortical language areas.

In contrast to the significantly higher right-lateralized activation observed in NH individuals in the present study, Giraud et al. [33] showed a left-lateralized activation of temporal and frontal regions in NH controls. However, direct comparison between the two studies is difficult as there are several differences in study design and implant experience of the participants. Mortensen et al. [37] found increased activity in right cerebellar cortex when running speech was comprehended relative to babble, but only in CI listeners with high speech comprehension. The authors speculated that this could be due to cognitive work of cerebellum subserving verbal working memory or a contribution of the right cerebellar hemisphere to precise representation of temporal information for phonetic processing. However, this finding was not replicated in the present study, which may reflect differences in duration of implant use and a mixture of speech comprehension levels.

### 4.2. Cross-Modal Plasticity

Giraud and colleagues [34] consistently demonstrated activation of areas BA 17/18 in the visual cortex when CI users responded to meaningful sounds. The authors argued that the process was associated with improvement of lip-reading proficiency, which is supported by findings in a behavioral study by Rouger [11]. A similar cross-modal interaction between vision and hearing was not replicated in the current study. Differences in the methodology used in the two studies may explain this discrepancy. The Giraud study involved repetition of words and syllables and naming of environmental sounds, contrasted with noise bursts, as opposed to the current study, which involved passive listening to a story contrasted with speech-like noise. Furthermore, sound was presented in free field, whereas in the current study, the auditory stimuli bypassed the microphones of the speech processor and were fed directly to the auxiliary input. Finally, the strict conservative statistical methods used here preclude reporting of results that are not statistically significant when corrected for multiple comparisons.

### 4.3. Limitations of the Study

In Scandinavia as in most European countries, cochlear implantation is administered by the public health care system and offered for free to all patients who meet the clinical implantation criteria (<40% open-set word-recognition scores). This includes patients with a prelingual hearing loss, despite recognition that these patients may have very limited linguistic benefit. As a result of this policy, the group of CI users in general is very heterogeneous, which was also the case in this study. While the difference in group size was far from optimal and may have confounded the results, we maintain that it is of high importance to also study cortical activity in prelingually deaf patients following cochlear implantation. In a previous study involving music training, we found that while little gain was achieved in speech perception, prelingual deafness did not preclude acquisition of some aspects of music perception [62]. This implies that plastic changes take place also in the long-term deaf brain and that specific training measures could help these patients in achieving improved implant outcome [58, 63].

Our normal-hearing control group had a less optimal size, which may have made direct comparisons between groups less valid than desired. However, ethical restrictions limit the number of healthy participants in studies involving PET.

As stated, the degree of deafness (i.e., residual hearing) varied across subjects and may have influenced the adaptation to the implant. Unfortunately, a correlation analysis between preoperative speech understanding and PET and behavioral results was not possible since such data were not available for all participants.

## 5. Conclusion

The present PET study tested brain activation patterns in a group of recently implanted adult CI recipients and a group of normal-hearing controls, who listened to speech and nonspeech stimuli. CI listeners with postlingual hearing loss showed differential activation of left superior temporal gyrus during speech and speech-like stimuli, unlike CI listeners with prelingual hearing loss. This group difference was also reflected in a behavioral advantage for patients with postlingual hearing loss. Furthermore, Broca's area was activated as an effect of time, but only in CI listeners with postlingual hearing loss. Comparison of the CI listeners and the normal-hearing controls revealed significantly higher activation of the caudate nucleus in the normal-hearing listeners. The study demonstrates that processing of the information provided by the cochlear implant is highly related to the history of hearing loss. Patients whose hearing loss occurred after the acquisition of language involve brain areas associated with speech comprehension, which is not the case for patients whose hearing loss occurred before the acquisition of language. Finally, the findings confirm the key role of Broca's area in restoration of speech perception, but only in individuals in whom Broca's area has been active prior to the loss of hearing.

## Figures and Tables

**Table 1:** Clinical and demographic data for the 15 participants included in the study.

Participant (gender)	Age at project start (*y*)	Etiology of deafness	Side of implant	^d^Pre/post	Duration of HL (*y*)	^ e^Degree of deafness (dB HL)	Implant type	CI sound processor	CI sound processing strategy
CI 1 (F)	49.8	^a^Cong. non spec.	R	Post	45.8	80–90	^f^Nucleus	Freedom	ACE 900
CI 2 (F)	21.4	Ototoxic	R	Pre	20.7	>90	Nucleus	Freedom	ACE 250
CI 3 (M)	31.7	Meningitis	L	Post	30.2	80–90	Nucleus	Freedom	ACE 900
CI 4 (M)	56.0	Cong. non spec.	R	Post	48.0	80–90	Nucleus	Freedom	ACE 1800
CI 5 (F)	70.3	Cong. non spec.	R	Post	30.3	80–90	Nucleus	Freedom	ACE 900
CI 6 (F)	47.5	Unknown	L	Post	10.5	80–90	Nucleus	Freedom	ACE 1200
CI 7 (F)	56.2	^b^Hered. non spec.	R	Post	37.6	80–90	Nucleus	Freedom	ACE 1200
CI 8 (M)	58.5	Meningitis	R	Pre	53.5	>90	Nucleus	Freedom	ACE 900
CI 9 (F)	29.1	^c^Mon	L	Post	19.1	80–90	Nucleus	Freedom	ACE 1200
CI 10 (F)	44.8	Unknown	R	Post	9.8	80–90	Nucleus	Freedom	ACE 1200
CI 11 (M)	60.4	Unknown	L	Post	16.4	70–90	Nucleus	Freedom	ACE 900
CI 12 (F)	50.6	Cong. non spec.	R	Pre	47.6	>90	^g^A.B.	Harmony	Fid. 120
CI 13 (M)	63.5	Cong. non spec.	L	Pre	57.5	>90	Nucleus	Freedom	ACE 500
CI 14 (F)	63.0	Unknown	R	Post	5.0	70–90	Nucleus	Freedom	ACE 720
CI 15 (M)	73.3	Trauma	R	Post	19.3	70–90	Nucleus	CP 810	ACE 720
Mean	**51.8 (SD 15)**				**29.7**				

^a^Non specified congenital HL, ^b^non specified hereditary HL, ^c^Mondini dysplasia. ^d^Pre- or postlingual HL. ^e^Measured as the average of pure-tone hearing thresholds at 500, 1000, and 2000 Hz, expressed in dB with reference to normal thresholds. Ranges indicate a difference between left and right ear hearing thresholds. ^f^Cochlear, ^g^Advanced Bionics.

**Table 2:** Main effects and interactions of analysis 1 performed across CI participants on the whole brain (top) and on a region-of-interest based on main effect of contrast (bottom).

	Coordinates			*Z* score	Region	Brodmann area
	*x*	*y*	*z*			
Whole brain analysis						
Main effect of contrast (RS versus BAB)	−58	−20	0	6.55	L STG	BA 21/22
	58	0	−6	5.67	R STG	BA 21/22
	64	−10	0	5.47	R STG	BA 21
Main effect of time					NS	
Interaction time x contrast					NS	
Main effect of group					NS	
Interaction contrast versus group					NS	
Interaction time versus group					NS	

Region of interest analyses						
Main effect of group					NS	
Main effect of time					NS	
Interaction contrast x group	−58	−26	0	4.09	L STG	BA 21/22
	−56	−16	−3	3.24	L MTG	BA 21

RS: running speech. BAB: babble. L STG: left superior temporal gyrus. R STG: right superior temporal gyrus. L MTG: left middle temporal gyrus, NS: non-significant.

**Table 3:** Main effects and interactions of analysis 2 performed separately for the two CI-subgroups on a region-of-interest based on bilateral inferior frontal gyri.

	Coordinates			*Z* score	Region	Brodmann area
	*x*	*y*	*z*			
POST HL subgroup						
Region of interest analysis (bil. IFG)						
Main effect of contrast (RS versus BAB)	−46	14	−6	4.91	L IFG	BA 47
	52	16	−6	3.85	R IFG	BA 47
	46	16	−9	3.74	R IFG	BA 47
Main effect of time	−42	20	9	4.29	L IFG	BA 45
Interaction contrast x time					NS	

PRE HL subgroup						
Main effect of contrast (RS versus BAB)					NS	
Main effect of time					NS	

L IFG: left inferior frontal gyrus. R IFG: right inferior frontal gyrus.

**Table 4:** Main effects and interactions of analysis 3 performed across CI- and NH-participants at baseline on the whole brain (top) and on a region-of-interest based on main effect of contrast (bottom).

	Coordinates			*Z* score	Region	Brodmann area
	*x*	*y*	*z*			
Cochlear Implant group versus NH group						
Main effect of contrast (RS versus BAB)	−58	−18	0	6.7	L STG	BA 22/21
	−54	4	−9	5.25	L MTG	BA 21/38
	62	−8	0	6.53	R STG	BA 21/22
	54	−44	36	4.64	R IPL	BA 40
Main effect of group	12	20	3	4.84	Caudate	
Interaction contrast x group					NS	

Region of interest analyses						
Main effect of CI versus NH (ROI)	58	−8	6	3.37	R STG	BA 22
Interaction contrast x group (ROI)	52	−46	39	3.45	R IPL	BA 40

L STG: left superior temporal gyrus. R STG: right superior temporal gyrus. L MTG: left middle temporal gyrus. R IPL: right inferior parietal lobule.
